# Supplementary material for: Splice-Junction-Based Mapping of Alternative Isoforms in the Human Proteome
Source: Cell Rep. Author manuscript; Available in PMC 2020 Jan 15. (PMC6961840; doi:10.1016/j.celrep.2019.11.026)

A

sp|Q14896|MYPC3_HUMAN|ENSG00000134571|SE2|23654|chr11|47347479|47347680|−2|r23|T1
TDSHEDTGILDFSSLLK q value: 4.1073e−05 Tr_novel:TRUE RefSeq_Novel:TRUE
Search result spec prec mz: 939.4634 Actual spec prec mz: 939.46343
Fragments matched per AA: 1.94 Proportion of top 20 peaks matched: 0.7

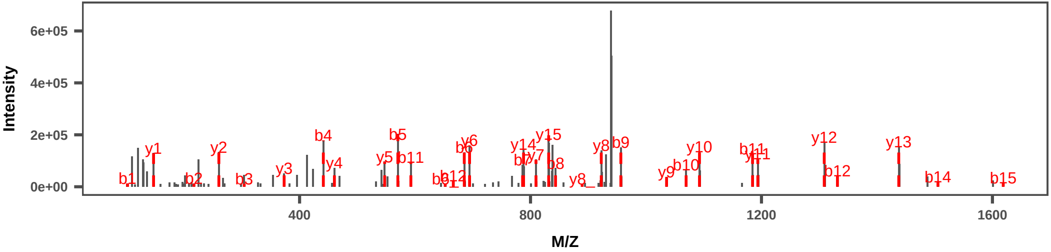

B

Scatterplot of predicted elution time
Fitting R2: 0.865
Novel peptide residual Z score: 0.709
Number of peptides: 1990

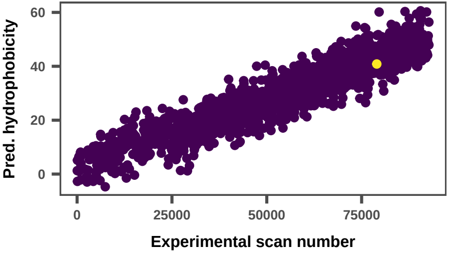

C

Distributions of residuals from best−fit line
of predicted RT vs Expt. scan number
Line: Z score of novel peptide
Z: 0.709

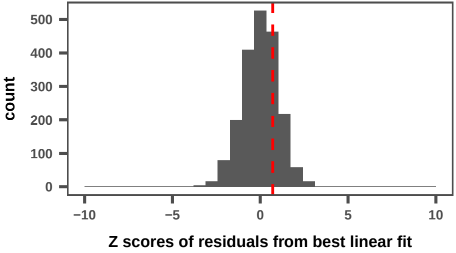

Supplement: 2 [file NIHMS1546469-supplement-2.zip › DF1/PXD006675/AtrialSeptum/AtrialSeptum_1_MYBPC3_TDSHEDTGILDFSSLLK.pdf]
